# The Mindful Way From Information to Knowledge, to Wisdom, and to Life: Perspectives on Mindfulness (-Based Cognitive Therapy) for Higher Education

**DOI:** 10.1007/s12671-025-02528-5

**Published:** 2025-02-18

**Authors:** Marc-Henri Deroche, Willem Kuyken, Teruhisa Uwatoko, Yuki Imoto, Ryotaro Kusumoto

**Affiliations:** 1 Mindful Living Research Group, Graduate School of Advanced Integrated Studies in Human Survivability, Kyoto University, Higashi Ichijokan 1 Naka-Adachi-Cho, Yoshida, Sakyo-Ku, Kyoto, 606-8306 Japan; 2 Department of Psychiatry, University of Oxford, Warneford Hospital, Warneford Lane, Oxford, UK; 3 University Health Center, Kyoto University of Education, 1 Fukakusa Fujinomoricho, Fushimi-Ku, Kyoto, 612-0863 Japan; 4 Department of Psychiatry, Graduate School of Medicine, Kyoto University, 54 Shogoin‐kawahara‐cho, Sakyo‐ku, Kyoto, 606‐8507 Japan; 5 Faculty of Science and Technology, Keio University, 4-1-1 Hiyoshi, Kohoku-Ku, Yokohama City, Kanagawa Prefecture, Japan

**Keywords:** Mindfulness, Higher Education, Wisdom, Attention, MBCT, Information Age

## Abstract

This article explores the potential relevance of Mindfulness-Based Programs, particularly Mindfulness-Based Cognitive Therapy (MBCT), to support the mission of higher education by facilitating the journey from information to knowledge, and from knowledge to wisdom. It thus addresses the problems of distractibility and superficial engagement caused by information overload and aims to prepare students for a fulfilling life. Based upon an in-depth dialogue among authors belonging to different disciplines, this conceptual synthesis integrates the various perspectives of Buddhist studies, philosophy of education, anthropology of education, clinical psychology, and psychiatry, to construct a comprehensive view of mindfulness for higher education. The structure of its argument progresses from the languishing to the flourishing of students, and from mindfulness taught in the form of interventions, to mindfulness cultivated as the very thread of learning. The article starts by reviewing the evidence regarding students’ mental health and vulnerabilities, and moves to directly listening to their voices, larger aspirations, and more existential concerns. It next elaborates an epistemic and developmental model of mindful education, making creative use of T. S. Eliot’s questions regarding information, knowledge, wisdom, and Life, to capture some ongoing, complex issues. MBCT’s principles, formats, practices, and adaptations are then examined to envision skillful responses to these perceived challenges, with a proposal to further weave mindfulness into the constitution of higher education. Ultimately, in reference to Simone Weil, mindfulness training is conceived as guiding the “formation of attention,” along the “joy of learning,” to accomplish two interrelated humanistic ideals: academic excellence and human flourishing.

Regarding the significance of mindfulness for education, Ergas (2019) has presented three approaches: mindfulness *in*, *as*, and *of* education. Mindfulness can be introduced as interventions—for example as programs for stress reduction or prevention of depression relapse. In such cases, the objective is functionalist and can be categorized as “mindfulness *in* education.” When mindfulness is reflected beyond the objectives of the intervention; for example, as a whole-school approach where the teacher, classroom space, and the whole institution embody mindfulness as a “culture,” this can be seen as “mindfulness *as* education.” When mindfulness becomes the radical catalyst for critiquing and re-envisioning education itself, Ergas terms this as “mindfulness *of* education.”

More recently, Roeser et al. (2023a) have built upon these distinctions, among many other valuable sources, to offer a much exhaustive and compelling vision for the next generation of science on mindfulness and compassion in schools for students. They suggested (p. 239) “(a) *improving* the experimental research; (b) *expanding* developmental research; and (c) *re-envisioning* assumptions, theories, and methods in research to go ‘beyond all splits’ towards a non-dualistic and relationally, culturally, contextually, ethically, and developmentally grounded science.” Roeser et al. (2023b, p. 235) insisted that “future interdisciplinary research that takes a lifespan developmental perspective could help the field to answer unanswered developmental questions that have value for experimental studies and educational practice.” In reference to Ergas (2019), the developmental approach by Roser et al. is thought to be organic rather than mechanistic, this perspective allowing to better envision mindfulness *as* education, and not only *in* education, or in other words: “mindfulness as a way of education and life, ethical conduct, and social transformation” (Roeser et al., 2023a, p. 245). Moreover, such an approach is considered necessary to improve experimental research in order to mind and bridge the gap between the enthusiasm for Mindfulness-Based Programs (MBPs) adapted to schools and the scientific evidence or experimental verification of their benefits (Felver et al., 2023, p. 279; Schonert-Reichl, 2023, p. 302; Weare, 2023, p. 294), as well as to enhance implementation of MBPs that are well-suited to the developmental condition and educational context of learners. Roeser et al. (2022) found that 52% of MBPs for schools included in their review were adapted from adult programming, raising the question of their fitness or relevance to school students (Baelen et al., 2023, p. 273), and calling upon advancing implementation science and practice in this field (Jennings, 2023). The fact that one of the largest cluster randomized trials of MBPs for schools and conducted in the UK (Kuyken et al., 2022) did not show the expected benefits of mindfulness (Boyce, 2022) has led to further emphasis on investigating the importance of contextual factors in implementation, highlighting the need for different research models and conceptualization. Various authors have also emphasized the foundational necessity to first train teachers in mindfulness, compassion, or social-emotional learning, before they offer such respective training to their students (Schonert-Reichl, 2017, 2023). Weare (2023, p. 297) writes that “this whole issue of teacher professional development needs to move to the front and centre.”

In their holistic and developmental model of education, Roeser et al. (2023a, p. 247) conceptualized “mindfulness as hypothetically developing through attuned interactions between a caring and mindful adult (e.g., a more knowledgeable other), a secure and curious child (e.g., the novice), and the cultural practice of joint attention in everyday life.” Thus, their “review suggests it is a good time to reflect and re-envision assumptions about how mindfulness and compassion might enter education *pedagogically* and *organizationally* in the most fruitful way (e.g., Ergas, 2019), and be studied in the most fruitful way” (p. 249). The way to teach students to be mindful seems first and foremost to have teachers themselves cultivate and embody mindfulness, so that they mindfully teach to, and relate with students with authentic presence.

Higher education is concerned with the education of young adults as well as older ones in recurrent education or lifelong learning, and forms thus a middle ground between children and teenagers who are in schools, and the world of adults. By rethinking education *with* mindfulness, and mindfulness *for* education, we intend thus to conceive a *mindful education* that not only constitute a body of information, but essentially consists in the very formation of attention, including joint attention—through vital pedagogical interactions—and ultimately in personal and social transformation developing during the entire lifespan. Indeed, higher education is preparing to a fulfilling adult life: it is the time of making major life decisions, choices that have an impact on the entire life. Loucks (2022, pp. 129–133) has skillfully proposed ways to use mindfulness in college “to clarify the best career path” by mindfully finding the alignment between personal aspirations and skills, with available jobs and opportunities.

Moreover, we feel here the further need to discuss developmental aspects with a closer attention to epistemological and phenomenological issues regarding the ways of knowing and being that are transmitted, formed, discovered, pursued, or even, for that matter, scientifically researched in higher education. This is especially important since evidence has been shown that the developments of children, teenagers, and young adults are strongly impacted by new information and communication technologies (Desmurget, 2019/2022). In our digital, and now Artificial Intelligence (AI) age (Kissinger et al., 2021), the incisive questions asked last century by T. S. Eliot (Choruses from *The Rock*, 1934) seem more relevant than ever. They may also offer heuristic categories to envision this developmental process, and hopefully reverse the loss of epistemological, ontological, and spiritual depth that was vertiginously perceived, and poetically expressed as follows:

Where is the Life we have lost in living?

Where is the wisdom we have lost in knowledge?

Where is the knowledge we have lost in information?

This citation has served to frame a thorough investigation of the question “how can/should higher education deal with the impact of the massive increase in easily retrievable information?” (De Corte & Fenstad, 2010, p. 1). Here, our main question is how can MBPs, and especially Mindfulness-Based Cognitive Therapy (MBCT) as adapted to the general population in higher education, facilitate to find the way from *information* to *knowledge*, from *knowledge* to *wisdom*, and from *living* on auto-pilot to *Life* lived in its fullness, along the educational progression and developmental maturation of a young adult in college/university? Such training, if well designed and implemented, is thus hypothesized to enable students to academically excel and to personally flourish before and after graduation.

MBCT (see Segal et al., 2013, first edition of the book published 2001) was initially developed by relying upon Mindfulness-Based Stress Reduction (MBSR; see Kabat-Zinn, 2013, first edition of the book published 1990) and combining it with Cognitive-Behavioral Therapy (CBT), with the aim to constitute a prevention treatment for depressive relapse. MBCT has since been extended to the general or healthy population: (1) in a slightly reduced format as “Mindfulness: Finding Peace in a Frantic World” (M-FPFW; see Williams & Penman, 2021, first edition published 2011) that is focused on relieving stress and has promising evidence in workplaces and education, or (2), in a format further developed toward well-being and flourishing, as “Mindfulness-Based Cognitive Therapy for Life” (MBCT-L; Kuyken, 2024). Well-being and flourishing have also been integral parts of similar interventions in higher education (Dvořáková et al., 2019; Henning et al., 2023; Hirshberg et al., 2022).

To offer a conceptual synthesis to respond to the research question, the authors of this article have tried to articulate the perspectives of their respective fields ranging from studying ancient wisdom traditions to advancing modern clinical sciences. This paper represents an effort “to navigate in the process of integrating paradigmatically different disciplines” (Crane et al., 2017, p. 997) around the transcultural and transdisciplinary concept of mindfulness in order to elaborate a construct of mindfulness that can be endowed with a shared meaning within higher education as a whole.

Ultimately, the article intends to further construct mindfulness training as potentially offering “*the* education *par excellence*” according to an often-cited quote from William James (1890, p. 424):

The faculty of voluntarily bringing back a wandering attention, over and over again, is the very root of judgment, character, and will. No one is *compos sui* if he have it not. An education which should improve this faculty would be *the* education *par excellence*. But it is easier to define this ideal than to give practical directions for bringing it about.

We shall see how the systematic “practical directions” for attentional control, emotional regulation, and self-awareness offered by MBPs, and MBCT in particular, may serve to implement such education *par excellence*. Technically, MBPs include (Crane et al., 2017, p. 990) the following:

A model […] of the causes of human distress and the pathways to relieving it; a new relationship with experience characterized by present moment focus, decentering and an approach orientation; […] development of qualities such as joy, compassion, wisdom, equanimity and greater attentional, emotional and behavioral self-regulation, […] sustained intensive training in mindfulness meditation practice, in an experiential inquiry-based learning process and in exercises to develop understanding….

MBPs adapted to higher education also have to negotiate the tension between self-knowledge and social engagement. To this aim, mindfulness cannot simply be reformatted according to mainstream or dominant culture, and thus such adaptation should offer a “mindfulness-based cultural critique,” so to speak, by questioning the *status quo*, and discerning alternative ways of being and knowing. Ethical and epistemological considerations are deeply intertwined in MBPs because beyond divisiveness or dualism, they consist essentially in the re-integration of body and mind, sensibility and conceptuality, emotion and reason, heart and head, self and other, humanity and nature, or, in sum, love and knowledge, as also compellingly presented by Roeser et al. (2023a) in their call to “go beyond all splits.” Here, we choose to keep the concept of mindfulness as a single umbrella or integrative term including compassion, since we understand mindfulness to refer to a *different quality of attention* that is intrinsically imbued with ethical attitudes such as befriending, compassion, joy, and equanimity (Feldman & Kuyken, 2019, pp. 144–182), to be indeed further cultivated by specific exercises that are generally parts of various MBPs, and explicitly and formally in M-FPFW and MBCT-L. We also construct mindfulness in closer and more explicit relationship to the philosophically overarching and transcultural virtue of wisdom (Deroche, 2021) that has also become the object of a psychological science for our times of polarization (Grossman et al., 2020).

## Methodology and Objectives

The present article intends to contribute to advancing the field by reconceptualizing the significance of mindfulness *in*, *as*, *of*, and *through* education (Ergas, 2019; Ergas & Hadar, 2019), and “beyond all splits” (Roeser et al., 2023a), by synthesizing various disciplinary perspectives around the relevance of one adult MBP, Mindfulness-Based Cognitive Therapy, especially because it has progressively been adapted for the general population, and with special reference to the lifespan: mindfulness *for* life, and here, *for* higher education. MBCT’s scalability to different formats makes it thus particularly relevant to higher education. Moreover, while a model of “second-generation” MBPs has been proposed with the aim to reassert Buddhist foundations (Van Gordon et al., 2020), our understanding of MBCT is that it is an ongoing integration of Buddhist wisdom and modern psychology. MBCT has been explicitly discussed in a cross-cultural and interdisciplinary manner by Feldman and Kuyken (2019), thus paving the path for the conceptualization of this paper. We seek here to offer a theoretical direction for a higher education that, through mindfulness, supports academic excellence and human flourishing. We also eventually provide practical suggestions for the use, adaptation, and design of MBCT’s psychoeducational content and meditative exercises in this context.

The process of writing this article started with the organization of a 1-day think tank on December 5, 2022, in a retreat format at Kyoto University’s Seifūsō (a traditional Japanese villa with garden), and focused on the topic of the potential relevance of MBCT for higher education in both East and West. It gathered 12 established scholars in Buddhist studies, cross-cultural philosophy, philosophy of education, anthropology of education, clinical psychology, and psychiatry. In this way, we intended to further deepen MBCT as an integration of ancient wisdom (Buddhist studies) and modern science (clinical psychology and psychiatry), adding between these two poles a series of mediations (cross-cultural philosophy, philosophy of education, and anthropology of education), eventually to advance the integrative, praxis-oriented knowledge that MBCT eventually constitutes according to our understanding.

In advance, each participant selected and shared with the whole group three research materials (mainly articles and book chapters) that they personally judged to be most essential readings in their own distinct discipline (including their own research) and most relevant to the pre-defined topic and agenda of the think tank. These pre-readings served to foster mutual learning, map the field, and establish common references among participants. The day included short sessions of mindfulness practice and was mainly devoted to an in-depth discussion structured into four sessions: “Mental Health and Well-Being at the University; Evidence-Based Research and Interventions; Culturally Sensitive Curriculum Design; Mindfulness as the Pillar of Education.” They were recorded, transcribed, and shared among all participants. Each of these sessions was chaired by one of the co-authors of this article, adopting thus, in rotation, the perspective of their respective academic field, each time with specified intentions. The day ended up with a general discussion and concluding reflections. After this expert meeting, a hybrid public symposium was organized at Kyoto University on December 10, 2022, with individual presentations by, and a panel discussion among, four of the authors who acted then as the representatives of the larger group. It then took eventually 2 years to expand our research and articulate our individual parts into this collective conceptual synthesis. The general editors of the journal, as well as the two anonymous reviewers, made substantial and valuable comments to further integrate our various elements into a structured argument, including further consideration of ongoing advancements in the field.

Through research readings and in-depth dialogue, sharing various experiences and reflections about teaching MBCT in universities, hosting public presentations and discussion, writing parts, and exchanging feedback, the systematic process of integrating our different disciplinary perspectives ultimately crystallized into the unified form of a fivefold argument. This single narrative progresses from the “languishing” to the “flourishing” of students, and from mindfulness *in* higher education (in the form of interventions) to mindfulness *as*, *of*, *through*, and *for* higher education, redefining its very mission and spirit:

1. We start here by reviewing the evidence regarding students’ mental health, and by considering their developmental vulnerabilities and the special care that these require from the college/university teachers, staff, and system.
2. We then direct our attention to listening to the voices of students, as they are alas too often muted. This serves to expand our reflection from mental health to examine the larger aspirations, and to some extent the more existential distress of students regarding studying, living, and the quest of meaning.
3. In this way, we elaborate on an epistemic and developmental model of mindful education, by envisioning the crucial role of mindfulness on the way back from information to knowledge, to wisdom, and to Life, addressing thus, one by one, the fundamental questions raised by Eliot.
4. We next inquire into how MBCT can facilitate such a journey. We identify MBCT’s core principles, various formats, practices, and possible adaptations, and make a simple suggestion to further weave mindfulness into the fabric of higher education, not as an extraneous element, but redefined as the guiding thread of learning.
5. From this perspective, we conceive mindfulness training for higher education as the very formation of attention, opening the way to, and finding flow through, the “joy of learning.” We offer thus reflections on this educational paradigm at the conjunction of two ideals: academic excellence and human flourishing.

## Mindfulness and Mental Health in Higher Education

### Special Care for Students’ Vulnerabilities

Students in higher education encounter a spectrum of vulnerabilities, extending from commonplace adolescent stressors to profound mental health disorders. The rising prominence of these challenges within academic institutions underscores the imperative to address them. Students with vulnerabilities may face more severe challenges, due not only to the pressure for academic achievement, together with uncertainties of identity unique to adolescence and anxiety about participating in society, but also to various difficulties brought about by mental disorders (Stallman, 2010). Students in higher education settings may face vulnerabilities arising from different types of mental disorders. Adjustment disorders, which are typically temporary and reactive; anxiety disorders, which may present by phenomena of social anxiety, panic attacks, and obsessive–compulsive disorders; and personality disorders, which arise from difficulties in the process of personality formation, can all contribute to these challenges. At the most severe pole, issues range from mood disorders to schizophrenia, which require intensive medical intervention (Lipson et al., 2022). Neurodevelopmental disorders such as autism spectrum disorders and attention deficit hyperactivity disorder further complicate the challenges by creating difficulties in establishing interpersonal relationships and managing attention (Gelbar et al., 2014; Sedgwick, 2018). In addition, the prevalence of substance-related disorders remains one of the most complex social issues to resolve (Skidmore et al., 2016). Often, these vulnerabilities manifest from academic challenges (Eisenberg et al., 2009). The failure to accumulate credits or complete tasks on time can be traced back to these underlying issues. However, beneath the academic struggles lie more profound challenges: a persistent struggle to focus attention, manage time, and forge meaningful relationships. As these accumulate, feelings of anxiety, frustration, and loneliness burgeon, potentially uncovering more severe psychiatric disorders (Campbell et al., 2022).

### Current Measures and Their Limitations

As inclusivity gains momentum in educational settings, there is an increasing push for environments conducive to students with varied challenges (Moriña, 2016). However, there remains a vast gulf between the ideal and reality. For example, reports and surveys from countries like the UK, the USA, and Japan reveal startling figures. In the UK, the percentage of students reporting mental health problems to their universities has exceeded 5% in 2020/21, almost seven times higher than in 2010 (Lewis & Bolton, 2023). However, the percentage is even larger in surveys conducted anonymously: a 2022 survey conducted by the mental health charity “Student Minds” found that 57% of responding students self-reported having a mental health problem, and 27% had been diagnosed with a mental illness (Student Minds & Alterline, 2023). In the USA, according to the Healthy Minds survey published in 2024 in which more than 100,000 students across 196 US campuses participated, 38% of students reported symptoms of depression; 34% said they experienced anxiety; and 13% said they were considering suicide (Healthy Minds Network, 2024). In Japan, a biannual survey on students’ mental health by the Japan Student Services Organization (JASSO) found that over 70% of undergraduate students feel anxiety about their future—about whether they will be accepted by their desired workplace or graduate school (Japan Student Service Organization, 2024). In addition, in a longitudinal study in 2021 of 985 university students, 17% had depressive symptoms, and 11.8% had suicidal ideation (Nomura et al., 2022).

Not only is there a high prevalence of mental health issues among university students, but there is also a considerable risk of their escalation as students progress in their academic journey (Grotan et al., 2019). Historically, universities have been sanctuaries of academic exploration, where the mutual exchange between mentors and mentees thrived under the ethos of academic collaboration. In these bygone days, the pace of learning was harmoniously aligned with the need for comprehensive understanding. Today’s landscape, with its accelerated drive for performance and outcomes, has dimmed this traditional glow, with vulnerable students feeling the most significant impact. Despite successes in diversity enhancement, there is a clear imperative for systems that not only offer reasonable accommodations for those with specific needs but also maintain the spirit of educational accommodation for all. Such a dual approach is vital for sustaining the mental and emotional well-being of the entire student community.

### The Institutional Context of Higher Education

Currently, an average university has a system of health services, counseling centers, and centers for assisting students with disabilities. For example, the health service focuses on early detection and appropriate referrals, the counseling center provides psychological support in university life, and the support center for students with disabilities is responsible for creating an inclusive university environment through reasonable accommodations (Duffy et al., 2019). However, in addition to these measures, a more organic approach is needed. For students who are aware of their mental stress, university interventions need to evolve. Effective interventions have the potential to transform the situation (Bantjes et al., 2022). It is crucial to tailor solutions to individual needs, not only to alleviate symptoms but also to enhance the student’s overall life.

Mindfulness training offers a timely orthogonal shift in the what and how of higher education. While early detection and medical interventions remain paramount, introducing mindfulness techniques can serve as an adjunctive approach. For students with chronic conditions or developmental challenges, mindfulness can be pivotal in identifying triggers, reducing stress, and preventing deterioration by increasing self-awareness of the body and mind and increasing resilience (Loftus et al., 2023). Furthermore, it can equip educators to understand better and support their students (Klingbeil & Renshaw, 2018; Schwind et al., 2011). For the average student, mindfulness can be a compass in navigating the tumultuous journey of university life. The benefits are manifold, contributing benefits to all students, including improved mental stability, increased aspirations, and a stronger sense of self-worth, and the effects benefit the lives of all students, not just those who are vulnerable (Galante et al., 2018; González-Martín, 2023).

However, incorporating mindfulness within university curricula is full of challenges. Resource constraints are palpable. Existing mental health centers, with their limited budgets, may find it challenging to spearhead such initiatives (Bantjes, 2022). Thus, specific efforts are necessary for the value of mindfulness to be recognized, adopted, and adapted by universities. Robust evidence, both academic and pragmatic, will be pivotal in realizing this vision. As higher educational institutions grapple with the escalating mental health crisis, embracing mindfulness can be a transformative step. It promises not just alleviation of symptoms but a holistic nurturing of the university community.

## Listening to the Voices of the Students

When we consider Eliot’s quote that established the central framework of this paper, sociological and anthropological approaches remind us that modern educational, institutional systems themselves of which we are a part, have contributed to creating the problems that they are now trying to alleviate. To reconnect with the “Life that we have lost in living” in and through higher education, there is a need to not only provide systems of care for students’ mental health and well-being, but to understand the perspectives and realities of the students as well as our own, the teachers and researchers. From this relational awareness of self and other, we can take steps towards mutual dialogue, and through that conversation work towards re-conceptualizing education and the ontology of “self” on which education is predicated.

Higher education is a continuously contested field but at its core—often muted—are the voices of the students. Thus, reflecting further on the previous section that outlined student vulnerabilities, we will consider how Eliot’s quote can be interpreted on the basis of the world experienced by university students themselves. Then, we review recent educational approaches that seek to respond to Eliot’s call of bringing Life back into education—specifically, the contemplative education approach that integrates knowledge and critical reflection with experiential learning. Mindfulness is the crucial vehicle in this integration process. In the USA, William Deresiewicz (2015) has fervently criticized the systemic and cultural entrapment of Ivy League education. In the case of Japan, educators have problematized the meritocratic culture that drives entrance exam competition, and that then leaves students in a moratorium after entering their goal of university. As we have discussed in the previous section, both developmentally and in terms of their liminal social status, university students are vulnerable to anxiety and psychological distress (Imoto, 2022). Below are comments left by undergraduate students at a first-tier Japanese university, taking a liberal arts anthropology class that incorporated contemplative approaches. Students wrote reflections and responses on an online class discussion board (November 2020), based on assigned readings about socialization. A first-year Japanese female student in the Faculty of Letters left this one:

Even if one day, I disappear from this society, there will be no problem for society at all. But to continue to live, we have to somehow work, and contribute to society, or else we will become subject to criticism. My heart/mind would not be strong enough to bear to live under such criticism. I sometimes wonder if I can become a human being that will be able to be of value to society. From junior high school up to my university entrance exams, I simply led life accepting the path laid in front, following the instructions of others.

There were many responses that followed from other students who had similar experiences and feelings, and who mobilized anthropology as a contemplative approach to re-envision how to live and the very meaning of life. A female student in Literature wrote: “perhaps by accepting yourself and finding a meaning to live from within yourself, it might be easier for you to live,” while a male student in Economics shared:

I also had a similar experience, I took entrance exams according to my parents’ wishes, and during lockdown, I thought about my future and how I can *become someone that contributes to society.* But what if we change the belief of what “society” is? Rather than thinking about whether we will be accepted and acknowledged or not by a large “society,” we can live the path that we choose to live. Society is made by me and others around me – there can be many kinds of societies. By thinking this way, perhaps we can live more happy lives.

Finally, a male student in the Faculty of Commerce wrote: “Just by being alive, we all have value, and we all have beauty and light, and this should be acknowledged without the need for any reasoning or rationalizing.” As such voices might indicate, students in this class experienced transformed views of society, culture and self, not simply through reading anthropological texts, but through reflection, contemplation and dialogue. Mindfulness practice, incorporated into such a class, enables students to become aware of their thoughts, to open to other perspectives, to reflect on the countless possible ways of knowing and being in the world, and to choose skillful ways of connecting with others and themselves.

It is useful to note here that education is based upon assumptions—or traditions—that may be radically different. The above quotes suggest that the dominant model of education and of society that the students have internalized is one that is functionalist: a view of education as socialization, where the role of education is to create subjects adapted to the efficient functioning of the nation state. The role of contemplative pedagogy is to move from a view of education as socialization to one in which our inner world has at least an equal footing in this process (Ergas & Hadar, 2021). In the case of MBPs, and especially MBCT, our proposal is that these are not only functionalist tools for mental health intervention, but that some practices (such as the Three-Step Breathing Space, as described below) start to permeate every aspect of student life, so that they become “mindfulness *as* education.” Furthermore, when coupled with critical reflection in the contemplative educational approach, mindfulness practice leads to a reconceptualization and liberation of self from limited worldviews. In some respect, institutions do constrain individuals and keep them living on learned automatisms. Ergas’ contemplative education theory guides us to be mindful not only in each moment, but to be mindful of our relations with others, and of the systems in which we are embedded and that may cause suffering. Elsewhere, researchers and educators such as Berila (2015) and Magee (2019) have also explored such creative use of mindfulness into their pedagogies.

Cross-cultural or anthropological approaches are useful in helping us to be more mindful of the underlying systems and cultural categories that we take for granted and that may constitute our blind spots. Reconnecting to *Life* requires not only the training of attention, but also an unravelling of an independent, separate sense of self, and the return to a sense of interdependent connectedness, and a sense of trust and friendliness towards the world based on an understanding of common humanity. This is something that can be learned, through common experience, critical inquiry, and scientific investigation (Center for Contemplative Science & Compassion-Based Ethics, 2019). At the same time, a re-envisioning of higher education would involve the sustained practice and education of the instructors themselves, so that all share and attend to each other’s presence within an ontology of interdependence from which compassionate awareness naturally arises.

## Towards an Epistemic and Developmental Model of Mindful Education: From Information to Knowledge, to Wisdom, and to Life

In this section, we suggest that Eliot’s questions can be seen as encapsulating the major challenges encountered and expressed above by students, and, as a form of *inquiry* (a concept that is central to MBCT; Segal et al., 2013, pp. 250–268), guide us to envision, in a structured response, an epistemological and developmental model of mindful education, or contemplative education, that can articulate together the different forms of knowing on the path of becoming a mature adult, of being fully human. This framework addresses what has been called the “cognition crisis” (Gazzaley, 2018) of our times, and highlights the concept of wisdom (on top of mindfulness and compassion), understood as the growing coordination of values and actions along the maturation of the lifespan (Piaget, 1965/1992). We shall now consider Eliot’s questions according to the sequence of information-knowledge-wisdom-life, to reverse the collapse felt by the poet, and find a mindful way through these various stages.

### “Where is the Knowledge We Have Lost in Information?”

The current information overload, exacerbated by the constant accessibility through ICT (especially smartphones), is leading to an increasing difficulty in selecting vital and reliable information from distraction and deception (Colon, 2023). As memory and attention are the two pillars of cognitive control, it is no surprise that academic performance has been observed to be negatively affected by the misuses of ICT (Gazzaley & Rosen, 2016). The overuse of screens tends also to reduce the scope of consciousness through diminished bodily and peripheral awareness, while absorbing and dispersing most focused attention or top-down attentional resources. Simultaneously, the constant solicitation of new stimuli overwhelms and captures bottom-up attentional resources through the mechanism of a surprise effect that, with its intermittent and addictive rewards, tends to lead to a mode of passive entertainment (Desmurget, 2019/2022). This way of attending to the world may be seen as the very definition of *distractibility*: narrow awareness (and thus low metacognition), unstable attention, forgetfulness of objectives, and therefore greater permeability to external suggestions or pressures, including propaganda.

The knowledge lost in information may also be understood as the decline of critical thinking or *concepts* due to the overflow of *images* (photos and videos) in current pathways of communication, together with their specific power to impress, suggest, or impose messages, by appealing to sensibility, emotion, and imagination, rather than to reason. A major consequence is also a form of de-realization: virtual appearances, perceived with an illusion of immediacy, are given more attention and interest, importance and value, than actual realities, themselves to be properly verified through careful examination, adequate categories, and sense-making (Larchet, 2016/2019, pp. 81–89). In this way, a weakening of linguistic abilities, from the richness of vocabulary, the complexity of grammatical syntax, to the capacities to read and write, has been noted. It has been even hypothesized than “iconic” thinking may also have partially replaced conceptual thinking (Larchet, 2016/2019, p. 114). The reliance upon screens, including television, has been seen as reducing the interactivity with parents and educators (Muppalla et al., 2023), and such a lack of dialogue may be hypothesized as reducing its internalization into proper reflection, as studies have shown the positive influence of dialogue on learning (Clark, 2023).

The knowledge lost in information is also the thread of the argument lost in separate bits of information. The culture of multitasking, brought by the design of computers, and its invasion of the classroom in higher education, with the temptation of immediately checking for extra references, further reduces the capacity of students to attend to the single organized narrative of a teacher (Jamet et al., 2020). Researchers in information theory and knowledge management referring to Eliot’s quotes have also further distinguished data from information (e.g., Bierly et al., 2000).

Larchet (2016/2019, p. 153) recalls the myth told by Plato in which King Thamus refuses the invention of writing offered by the god Theuth. The King considers that it would impair the faculty of memory, and given the central importance of the latter in the context of a then largely oral culture, writing would damage the overall edifice of knowledge and the very relation to truth (Plato, 1997, *Phaedrus*, 274–275). Thus, the dilemma of epistemic gains versus losses with the advance of technology is certainly not new. Yet, like for Plato who, despite mentioning these concerns, eventually chose to write down the oral dialogues attributed to Socrates, this problem requires from us historical perspective, epistemological reflection, and educational strategy.

Critical reflections on our values system are especially important to overcome current challenges related to the economization of attention in the digital evolution of capitalism, according to which profit is gained from online viewers by the way of sophisticated techniques that grasp and keep hold of their attention. Mindfulness, in its philosophical and ethical depth, means to question and reframe, rather than simply to assist these new forms of exploitation and alienation (Hyland, 2016; O’Donnel, 2015; Reveley, 2015).

### “Where is the Wisdom We Have Lost in Knowledge?”

The effort that is necessary to obtain reflective knowledge, literally of critical importance, may, as we have just discussed, be impaired by the addiction to the immediate gratifications derived from the exciting consumption of digital information. But the next issue is that, in modern education, such knowledge may remain solely conceptual, and not engage the whole person, mind and body, reason, and emotion. Such learning may therefore ultimately reinforce the general tendency of “living in our heads” (Segal et al., 2013, pp. 144–176), an expression referring to the state in which consciousness is mainly absorbed into thoughts, or re-*present*-ations, rather than attending to the present experience. Since this condition may deviate into rumination (a major mechanism common to depression and anxiety), or even into dissociation, an education that is overly conceptual may not prevent such mental disorders; it can even further exacerbate them.

The distinction between conception and perception, or two ways of knowing, through an indirect representation, or a direct, embodied, present-centered awareness, has thus been articulated in MBPs with the notions of the “doing mode” versus the “being mode,” while evidence of two correlated cortical networks of self-reference has been shown (Farb et al., 2007). Both modes are eventually complementary (Feldman & Kuyken, 2019, pp. 124–126), but one’s sense of being may get lost in overdoing and striving to a goal, while from the ground of a deeper presence may emerge an action that is better suited to the larger context. MBPs offer thus a new approach in education, by rebalancing both aspects, and especially training and refining various forms of direct perception.

*Knowledge* may also refer to purely cognitive skills, while *wisdom* may encompass socio-emotional skills as well. It may be useful to mention that this distinction actually reflects a long Western legacy, with Aristotle’s twofold philosophical psychology exposed in *Nicomachean Ethics*, distinguishing “intellectual virtues” from “moral virtues,” each category belonging respectively to the rational and the emotional parts of the psyche (1996, I, 1103a4–1103a13). From this historical and philosophical perspective, social and emotional skills are identified as *moral virtues*, and are thus, per definition, *ethical*. The highest intellectual virtue was conceived as *sophia*, theoretical or contemplative wisdom (culminating in a direct *vision*, and the love of which defined the very project of *philosophia*), while the highest moral virtue (coordinating all others such as temperance and courage, etc.) was *phronesis*, practical wisdom based upon life experience. It is thus possible for us today to construct wisdom as integrating both cognition and emotion. Social-Emotional Learning and related initiatives were indeed skillfully developed in response to the perceived hypertrophy of cognitive skills, and the concomitant atrophy of social-emotional skills in modern education (Greenberg et al., 2017). In the end, academic *knowledge*, if limited to a purely scholarly understanding, is radically different from the personal possession of virtues, or *wisdom* in the largest sense.

### “Where is the Life We Have Lost in Living?”

The previous elements point out the need for a holistic education including life skills, a *savoir-vivre* that could provide resources to personally answer the perennial questions: “how to live?” and “what is the good life?” We may interpret the *Life* that has been lost in *living* as consequence of the exclusive focus of modern education on the acquisition of future social status and wealth in order *to make a living* (which is, of course, a necessary aspect of life), and thus losing sight of the *Life* that is* worth living.*

From the perspective of MBPs that, according to their Buddhist sources, place great emphasis on the exercise of mindfulness of the breath (Pāli: *ānāpānasati*) (Kabat-Zinn, 2013, pp. 39–53; Segal et al., 2013, pp. 182–184), the *Life* that is lost in *living* may be understood as *living* without knowing that one is living, especially that one is breathing: not being aware of the very breath of *Life* that animates the whole body and mind, and that flows rhythmically back and forth through the individual and the natural environment. Mindfulness training teaches thus in a sense “not to forget to live” (Hadot, 2008/2023) and to remember to breathe. The lost *Life* may also be seen as the disconnection from the “life-world” (*Lebenswelt*) as elucidated in phenomenology (e.g., Husserl, 1936/2006), the *Life* that is directly felt from within. Ultimately, *Life* may refer to the fundamental *joy of living*, the joy of *simply being alive*, content to just be (Hadot, 1995/2002, pp. 194–198; Williams & Penman, 2021, p. 31). And this “point at which life rejoices in life is precisely the present instant” (Hadot, 2008/2023, p. 37). It is such *Life* that is eventually to be recovered by the practice of mindfulness.

### The Mindful Way from Selecting Information (➤ Studying) to Organizing Knowledge (➤ Reflecting), to Embodying Wisdom (➤ Practicing), Back to Life

As it has already been proposed in the preceding sub-sections, MBPs offer important resources in order to answer each of Eliot’s three questions. We shall now connect these different dots to present an overall figure of mindfulness that can form a response to the current challenges thus perceived in higher education, on the way back to *Life*, and for a more viable civilization.

First of all, mindfulness (Pāli: *sati*; Sanskrit: *smṛti*) is traditionally defined in Buddhist sources as the mental factor that has the very function of “non-distraction” (Asaṅga, 2001, p. 9). It may thus be reasonably constructed as the core antidote to the root cognitive problem of the information age: distractibility. MBPs, as we shall see below with special reference to MBCT, offer a systematic training to re-balance top-down and bottom-up attentional systems, cultivating both a stabilized attention and a broadened awareness (and therefore meta-awareness; Sumantry & Stewart, 2021; van den Hurk et al., 2010). Such mindfulness-based re-equilibration forms the foundation for all learning purposes.

Mindfulness can then be further envisioned as the very pillar of learning, paving way from information to knowledge, to wisdom, and to Life. This requires of course to define mindfulness beyond the limited time spent in formal meditation, and to understand it to dwell at the crux of both conceptual and experiential modes of knowing, as the capacity to flexibly alternate and integrate them (Feldman & Kuyken, 2019, pp. 59–64).

Today, in search of such a model, it may be relevant to refer to the original framework found in Buddhist sources, according to which the integration between rational discourse and meditative experience was elucidated along the developmental path of wisdom, and mindfulness acting as its very thread (Deroche, 2021, p. 20). Early and especially Mahāyāna Buddhist texts and traditions describe the development of wisdom as progressing, based on ethics, from (1) “the wisdom born from studying” (literally: from “listening,” but also including reading and memorizing) to (2) the “wisdom born from reflecting,” to (3) the “wisdom born from practicing (formal meditation)” (Vasubandhu, 1990, VI, 5a–b). These three steps in the development of discernment or wisdom thus articulate dynamically different epistemic sources, or ways of knowing, corresponding respectively to (1) the authority of tradition (teachers and texts), (2) the rules of logical reason, and (3) the act of direct perception, or intuition in its various forms, especially enhanced by meditative concentration (Anālayo, 2003, pp. 44–46).

Then Vasubandhu (1990, VI, 15a) also defines the essence of the application of mindfulness as being this threefold wisdom (Anālayo, 2021). Mindfulness can thus be envisioned as *presence* according to the progression of these three stages of wisdom (Deroche, 2021, p. 29): (1) keeping what has been learned *present* in mind, (2) formulating adequate re-*present*-ations, and (3) cultivating a *presence* of mind, ultimately resting in the very *presencing* of lived experience, of pure awareness. This model also serves to integrate the various and sometimes contradictory relationships of mindfulness with memory, judgment, and attention (Gethin, 2015, pp. 33–34), these three elements being at the core of all cognitive operations in education, and particularly exemplified in memorization, critical thinking, and active engagement (directing and sustaining attention) in the process of learning.

The relevance of this model to modern education has been elsewhere examined (Klebanova, 2022; Kusumoto, in press). Our thesis here is that it can offer a coherent response to Eliot’s questions. The integrative path of mindful wisdom progresses (1) through the wisdom born from study, from selecting and retaining vital *information*; (2) through the wisdom born from reflection, to organizing *knowledge*; and (3) through the wisdom born from practice to embodying *wisdom*, in order, ultimately, to *living* our lives in re-alignment with the deeper awareness of *Life* itself. In sum, such is the way from the *information we get* from outside, to the *knowledge we have* established within us, to the *wisdom* (that is inseparable from who) *we* (truly) *are*. By closely linking the concept of mindfulness to that of wisdom, as conceived in East (Sanskrit: *prajñā*) and West (Greek: *sophia*), we also wish to reassert a humanistic and cosmopolitan ideal for education as the formation of a moral agent in age of distraction (Crawford, 2015), and to further strengthen what has also been referred as “cyber-wisdom” (Polizzi & Harrison, 2022). Let us see now how MBCT could contribute to implement such a path in higher education.

## The Relevance and Adaptation of MBCT to Higher Education

### Various Scales and Formats

One of the central questions for this paper is how MBPs, especially MBCT in its adapted form in higher education, can facilitate the path which we have explored above. Crane et al. (2017) articulated how there are essential components to what define MBPs (the “warp”), but there are also differences (the “weft”) that are employed “to make the mindfulness framework and practices more accessible and useful for particular populations and in varying contexts” (p. 996). While there are numerous forms of MBPs, the value of any approach lies in how easily it can be accessed by those who could benefit from it. There is a need to find ways to enhance the accessibility of MBPs and make sure that they constitute a path that individuals can choose to follow in their lives, adapting it to their needs and for as long as it benefits them. Thus, this section will explore how existing contents, formats, and scalability of MBCT programs can be transferred and weaved into the fabric of higher education for the people within the institutions (students, teachers, and staff), as different courses, with different formats, for different scales. Ultimately, the aim is to bring the essence of MBCT not as a simple additive for education, but as a vehicle that supports the continual path from information, to knowledge, to wisdom, and to Life.

Scaling is an important process to enhance accessibility. Scaling of MBPs can be understood as a funnel. The opening of the funnel is where a larger portion of the population might first be introduced to mindfulness. This will be in their homes, schools, and workplaces, where other people embody the aforementioned qualities, and mindfulness is learned through them. As we move into the funnel, there is the opportunity to undertake a more intensive training through the 8-session courses, which is then followed by more advanced courses. The funnel provides a pathway for learning mindfulness, where earlier entry points are more accessible, less demanding, and courses later in the pathway require more of people but are also potentially more transformative. We will explore different formats of MBCT from the narrowest end of the funnel to the widest, thus showing how MBCT can be integrated into higher education increasingly to the wider population, eventually suggesting how it can shift the culture of higher education itself.

The aforementioned MBCT-L program is considered a standard program which can be developed on or scaled into various formats. The 8-week MBCT-L course is a group-based teacher-led training intended to support participants’ learning. MBCT-L requires a well-trained teacher and is offered in groups with a cap of typically 15 participants. There is promising evidence of its effectiveness and acceptability to people who are interested in such an approach and have the resources and time to commit to it (Strauss et al., 2021). Many people who take part in MBCT-L find it helpful, and even transformative, and want to continue to deepen and extend their learning. Such needs have been met in “Mindfulness – Taking it Further” (MBCT-TiF) which consists of 12 sessions of 120 min each. When compared with sustained mindfulness practice, MBCT-TiF has been found to improve psychological quality of life along with symptoms of anxiety and depression (Maloney et al., 2024). Similarly, “Deeper Mindfulness: Exploring Feeling Tone Frame-by-Frame” (Williams & Penman, 2023) has also been conceived as a program for graduates of standard MBPs, shown to have similar positive effects (Williams et al., 2022); the program runs in eight sessions, 120 min each.

Given its extensive format, these courses are relatively intensive interventions and will only ever be pertinent to a relatively small part of the population. However, that small number of experienced and motivated people can act as a linchpin for the culture of mindfulness in the university. Especially, as others have suggested (Barbezat & Bush, 2014, pp. 67–68; Shapiro et al., 2016), teachers’ depth of experience with the practices and their embodiment can become a foundation for establishing an environment conducive for cultivating mindfulness within the classroom, just as how the training level and experience of MBP teachers affect participant experience (Ruijgrok-Lupton et al., 2018). For the students, these programs may best be adapted as an extra-curricular, non-credited activity, as each session of these programs take longer than the typical length of a university class.

If we consider implementations above as positioned in the narrowest end of the funnel, a MBCT family program designed for a wider audience is “Mindfulness: Finding Peace in a Frantic World” (M-FPFW; Williams & Penman, 2021) that is focused on relieving stress and has promising evidence in workplaces and education. M-FPFW training consists of eight sessions of 90 min each and thus fits well with the context of higher education as it can be easily integrated as part of a regular course of 15 sessions of 90 min, a standard in academia, and offering credits to registered students. Moreover, the home practice recordings are shorter in length compared to MBCT-L (each no more than 15 min) and are thus better tailored for the schedule and capacity of students. The effectiveness of the program for both teachers (Beshai et al., 2016) and students (Galante et. al., 2018; Medlicott, 2021) has already been evaluated. MBSR has also been similarly adapted into a format fitting for college class schedule and has shown to improve student overall health and self-regulation (Loucks et al., 2021). Other related short mindfulness programs for university students have also shown promising results (Vorontsova-Wenger et al., 2022). Integrating mindfulness in for-credit classes should be done carefully so as not to position the practices themselves as an evaluated homework; when students are under such impression, the practices are transformed within each student as something they *must do* for credit, thus reversing their entire purpose. At all levels of implementation, negotiation with, and awareness of, students’ motivation for practice is paramount. As pointed out by Barbezat and Bush (2014, p. 69), ignoring (or being unaware of) the level of resistance by students may lead them to deeming that “their experience actually does not matter,” and thus they “may not believe the stated intention of the exercise.” As shown by Sevilla-Liu et al. (2020) in their work analyzing phenomenological descriptions of graduate students’ experiences with MBCT, each student may experience the program differently; teachers should be aware of such differences to deliver the program in a helpful and safe manner.

There are also benefits to offering them within for-credit courses, including access to the environment constructed by the learnt teacher. Peers can also support a sense of shared accountability to engage in the content and activities, co-creating the learning environment with the teacher. Of course, M-FPFW and MBCT-L can be offered as non-credit extra-curricular programs as well, including more intensive formats such as a multiple-day workshops or residential programs.

As M-FPFW and MBCT-L are still 8-week courses (possibly 6 for M-FPFW), it will be limited to students interested enough to commit themselves to such a class. For those interested but not ready for such intensive programs, the Oxford Mindfulness Foundation, among others, offers “Introducing Mindfulness” which consists of three sessions, 60 min each. Adaptations of this program can be transferred into for-credit classes, or we can also conceive a 3-h, intensive extra-curricular workshop. The effectiveness of similar forms of short programs have been evaluated (Howarth et al., 2019), even specifically for students (Shearer et al., 2016). Mindfulness can also be introduced to any classroom (with a competent teacher) as a short, 5-to-10-min practice at the beginning and/or end of class. Studies have pointed to the possibility of how even a single short practice can improve cognitive performance including allocation of attentional resources (Johnson et al., 2015; Norris et al., 2018).

At the next section of the funnel lies introduction to mindfulness though apps and books. Mindfulness has been made more broadly accessible than ever before through these mediums. But research on mindfulness apps suggest that for the vast majority, such access to mindfulness is short-lived (Flett et al., 2019; Linardon, 2023). Also, it offers a limited understanding of mindfulness, and an understanding of mindfulness practice as something someone listens to on one’s phone. Learning mindfulness through apps can be especially challenging as there is a plethora of apps that does not meet the standard of quality (Schultchen et al., 2021). Simply put, accessibility should not be a trade-off with integrity.

Thus, when we scale MBCT into higher education, adapting the format for each context, a sense of integrity and fidelity must be maintained. In other words, the core principles of the MBCT must be identified and contained; only from that standpoint can a flexible adaptation arise. The “Introducing Mindfulness” course, which is the most essentialized structured course within the MBCT family, identifies three core themes: (1) paying attention, on purpose, with curiosity and care; (2) perspective: learning new ways of being; and (3) responding skillfully. Session 1 covers how attention is a gateway to experience, and thus places training of attention (and attentional flexibility) as the central objective. Session 2 explores how that trained attention can free us from the entrapment of habitual thinking mode, and opens the door to experiential mode; the enlarged field of awareness acts as a space between stimulus and response. Recognition of these two modes prepares the ground for the theme of session 3, which is to train our capacities to respond to challenging situations from this experiential mode, rather than to react habitually and emotionally. Thus, the core themes of MBCT exemplifies a mode of cultivating attentional control, self-awareness, and emotion regulation. The different possible adaptations into higher education listed above must thus contain such essence.

Finally, as we have already noted, the prevailing context, culture, and climate in which we grow up, study, work, and live powerfully shape our lives. We would like to suggest that when these are imbued with qualities of awareness, curiosity, kindness, care, and resilience, the people within such environments flourish. This evidence for these broader influences, especially that of institutional, school culture, on mental health and flourishing, is compelling, but has not been much explored in the field of mindfulness. To enact mindfulness *as* education means that there is a cultural shift within the institution to one that incorporates the “mindfulness language” rather than the “mindfulness intervention” (Ergas, 2019, p. 350). This shift does not happen when mindfulness is only considered something *apart* from the educative process, as something supplementary; the “mindfulness language” must be imbued from within existing classes. In this section, we have explored how the principles of MBCT can be integrated into higher education at different scales, in different formats. In the next section, we will focus on the Three-Step Breathing Space and explore how it can be a central practice that weaves mindfulness into each existing classes of higher education, as the heart of learning itself, serving as a foundation to develop mindfulness as the larger culture of university.

### Weaving Mindfulness into the Constitution of Higher Education: The Thread of the “Three-Step Breathing Space”

A simple proposal of the present article is to integrate into the university classroom the seminal exercise of the so-called Three-Step Breathing Space (3SBS) of MBCT. The rationale is that it requires only a few minutes (schematically three) but when done at chosen intervals (especially at the start of a class), it can change the entire course flow of teaching and learning; other educators and researchers have found that even a few minutes of silence at the beginning of class can make students more present (Barbezat & Bush, 2014, p. 39). Then, arguably, 3SBS condenses the essence of MBCT’s family of curricula (Feldman & Kuyken, 2019, pp. 126–127; Segal et al., 2013, pp. 383–390), and as such can serve as an introduction (or invitation to learn more for beginners); as a summary (or reminder for those who have previously been trained); and fundamentally as a seed planted in the soil of higher education that is a way of integrating mindfulness in its culture.

**Fig. 1:**
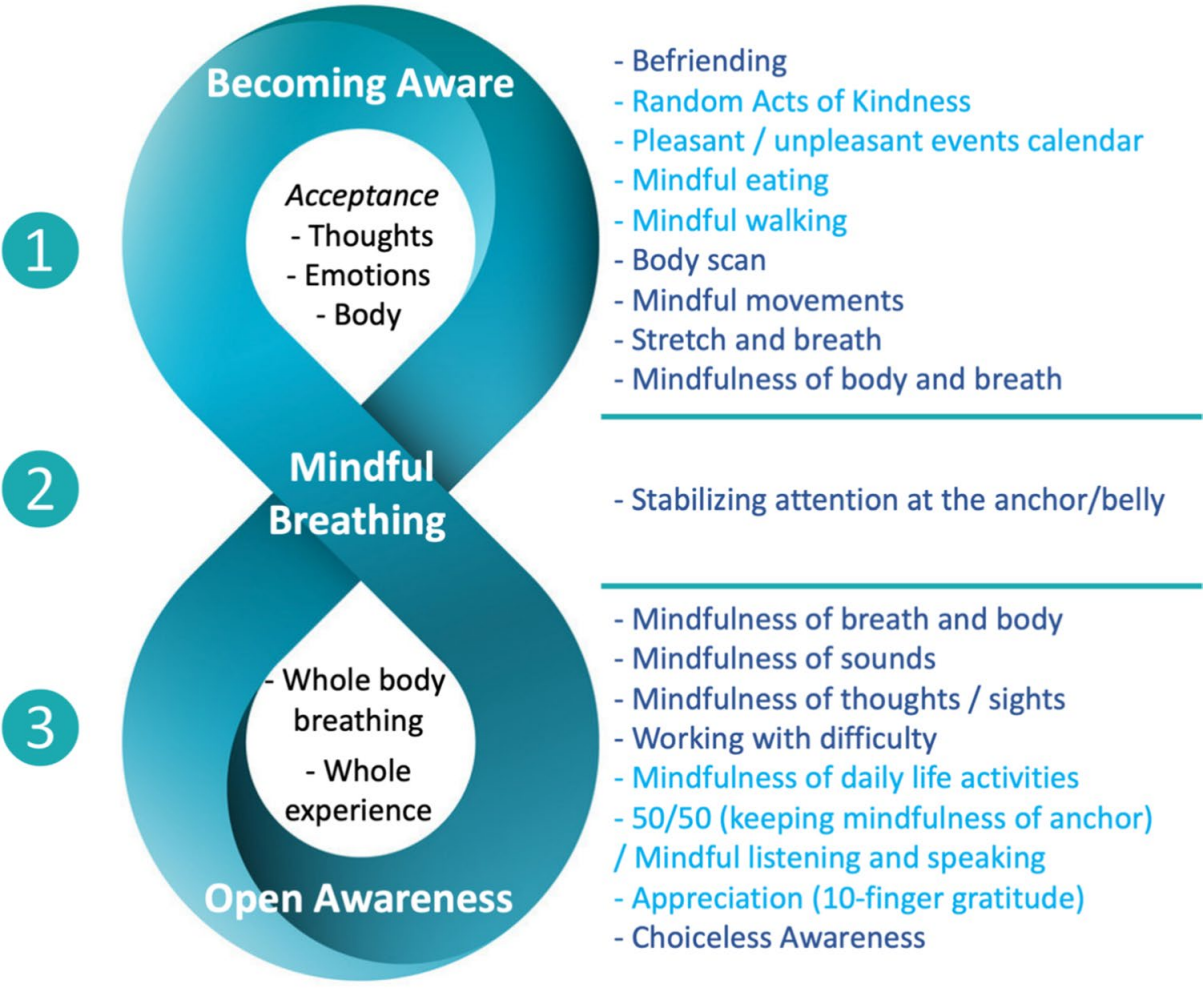
The Three-Step Breathing Space as condensing MBCT-L practices

Looking into the structure of the 3SBS also allows us to consider the relevance and adaptation of MBCT to higher education, by identifying its essence, making sense of its different elements, of their specific purposes and overall interconnection, so that it is then possible to skillfully weave them into the fabric of academic life, coping organically with its particular circumstances. In this way, it is possible to group (as shown in Fig. 1) the psychoeducational contents and mindfulness practices of the 8-week course of MBCT-L (taken as an example) and the skills that they serve to cultivate, according to the threefold process of the 3SBS.

Step 1, “Becoming Aware,” consists in deliberately enlarging awareness, becoming aware of thoughts, emotions, and bodily sensations, stepping back from automaticity, and approaching them with acceptance and careful interest. This short self-recollection synthetizes the elaborate inquiry, central to MBCT’s integration of Buddhist psychology and CBT, that distinguishes in any conscious experience the various facets of context, thoughts, feelings, sensations, and impulses (Feldman & Kuyken, 2019, p. 102; Segal et al., 2013, p. 307), and frees the mind from being entangled with them in vicious circles of psychological distress, as this does happen when they are not discerned clearly due to a lack of awareness and the co-emergent drive of subconscious reactivity. Moreover, the fundamental spirit of acceptance that underlies the practice of mindfulness is especially enhanced by the practice of befriending and compassion, of opening and turning toward experience, as it is.

Step 2, generally labelled as “Gathering” (Feldman & Kuyken, 2019, p. 127; Segal et al., 2013, p. 208), consists then in progressively narrowing the field of awareness, focusing on one chosen bodily anchor, and grounding, or stabilizing attention there. This resting place for the mind may vary according to individual needs, the care for past traumas, mental agitation, or highly stressful circumstances, and involve the parts of the body that are in contact with the floor or seat, the feet, the hands, or the lower abdomen (Treleaven, 2018, pp. 120–122). But in all cases, using the breath as a vehicle for establishing awareness in this location is central. Thus, Step 2 is termed in the figure below as “Mindful Breathing.” If one chooses to focus more exclusively to the breath, it may then be sensed predominantly either in the abdomen or at the nostrils. Again, mindfulness of breathing is paradigmatic for MBPs, as it brings a therapeutic re-unification of body and mind, self-soothing, and revitalization (Kabat-Zinn, 2013, pp. 37–52).

From this firm foundation, Step 3, generally labeled as “Expanding,” consists in gradually re-enlarging the scope of awareness to include the sensations of the whole-body breathing, breathing mindfully while being aware of the entire body, and then embracing the totality of experience with “Open Awareness,” as we choose here to refer mainly to this step. The practitioner may finally reach choiceless awareness, in a sense the heart of mindfulness practice, and from which a compassionate responsiveness may naturally emerge.

The 3SBS is also taught using a visual design, generally an hourglass shape (Feldman & Kuyken, 2019, p. 126), that reflects the oscillations of the aperture of awareness along the short time duration this exercise takes, from open to narrow, and from narrow to open again. This representation can offer a simple pedagogical method to transmit to students the essential psychoeducation on attentional control, self-awareness, and emotion-regulation, in a way that is accessible, memorable, and applicable. But since in the context of higher education, such exercise is mainly meant to establish the presence of mindful awareness for better engagement into the process of learning, and to do so, maintaining the continual flow of mindfulness is essential. Thus, we suggest here the possibility to use an eight-shape figure of the 3SBS (Fig. 1). Inspired by Arthur Zajonc’s (2009, pp. 39, 41) infinity symbol-shaped illustration weaving focused attention and open awareness, this model can thus serve to visualize the constant application of mindfulness in study and in daily life (including in so-called informal practices of MBCT). Evoking as well the undulating movement of the breath of life—the awareness of which dwells at the core of the model—the shape of the eight indicates the capacities of periodically re-enlarging perspective and checking in, and then focusing more exclusively on a given object or task, thus rebalancing bottom-up and top-down attentional systems, to eventually stay with both stable and open awareness, from which next to respond mindfully, with eyes wide open, to the various calls for attention and care, problem-solving and efficient action.

In the words of the philosopher Simone Weil (1950/2018), “forming the faculty of attention is the true goal and almost the unique benefit of studying” (p. 85, *la formation de la faculté d’attention est le but véritable et presque l’unique intérêt des études*). But, “In order to really pay attention, one must know how to do so” (p. 99: *Pour faire vraiment attention, il faut savoir comment s’y prendre*). MBPs, particularly MBCT and its condensation, the 3SBS, offer precisely such a practical know-how for our times. Eventually, attention is for Weil a “negative effort” (p. 104), because “Attention consists in suspending one’s thinking activity, leaving it available, empty and penetrable by the object” (p. 107: *L’attention consiste à suspendre sa pensée, à la laisser disponible, vide et pénétrable à l’objet*….). The 3SBS offer a didactic way to such a receptivity, and preliminary requisite for studying: being *aware*, *mindful*, and *open*.

## Rediscovering the Joy of Learning Through Mindfulness

As it has been bitterly discovered in the MYRIAD study, school students did not fully participate in the formal practices of mindfulness (especially, they did not perform home practices; Kuyken et al., 2022). So here, in the context of higher education, rather than to teach mindfulness as a separate curriculum that may only interest smaller groups according to different scales, our suggestion is to teach the majority of university students how to rely upon mindfulness in order to live and study well. The last main argument of this article is that they may be better motivated in this way, since such an approach would meet them where they currently are, and from there help them to rediscover a deeper form of joy and curiosity in the process of learning.

Contrarily to some contemporary representations of MBPs, Weil (1950/2018) warned: “Most of the time, attention is confused with a kind of muscular effort” (p. 99: *Le plus souvent on confond l’attention avec une espèce d’effort musculaire*). According to her observations, although willpower plays an important role in directing effort, she writes: “Intelligence can only be led by desire. For there to be desire, there must be pleasure and joy” (p. 102: *L’intelligence ne peut être menée que par le désir. Pour qu’il y ait désir, il faut qu’il y ait plaisir et joie*). She also considered two types of attention, (1) a discursive and inferior type of attention applied to school matters that, if well exercised, could allow the emergence of (2) an intuitive and elevated type of attention, leading in her understanding to contemplate God, or we may say here with Eliot, *Life* itself. Thus, for her, through the medium of attention, study was to be ordinated toward contemplation. And a contemplative approach to attention in education was for Weil relying upon “the joy of learning” (p. 102: *La joie d’apprendre*). The ultimate purpose of education was conceived as the formation of attention: “Happy then those who spend their adolescence and youth only to form this power of attention” (p. 116: *Heureux donc ceux qui passent leur adolescence et leur jeunesse seulement à former ce pouvoir d’attention*).

Nowadays, MBCT could thus contribute to the elaboration of such a formation or *Bildung* (Weare, 2023, p. 294). For most students, on top of the 3SBS, another major resource is the framework of the “five hindrances” (Feldman & Kuyken, 2019, pp. 137–138) and the correlated “five absorption factors.” We concur here with Anālayo (2022) who has argued for introducing these categories in the context of integrating mindfulness into education. Although initially developed in the Buddhist context of in-depth meditative absorption practice, this framework proves to be very useful when applied to the experience of a student in higher education. Many assignments must be carried out alone, and solitude forms a very challenging situation for young adults whose executive functions have not entirely matured (Tervo-Clemmens et al., 2023).

As astutely described by Gethin (1998, pp. 180–181), the first obstacle when starting an academic task consists in the tendency of wanting to do something else that may provide immediate pleasure or relief. This is “sensual desire.” The second obstacle is then reluctance or “ill-will” to perform the task, leading to procrastination. The third one is the drop of energy level, “sloth-and-torpor,” or, in other words, feelings of fatigue and depression. The fourth one is at the opposite end of the spectrum: it is “restlessness-and-worry,” or agitation and anxiety. Ultimately, the most subtle is “doubt,” the lack of a clear decision and intent that can jeopardize the whole enterprise of learning. Recognizing these hindrances, one can then rely on supportive factors to rebalance psychic energy, in sum: finding flow beyond the two extremes of boredom and anxiety (Csikszentmihalyi, 2008, p. 74).

The first two factors of absorption are often translated as “directed attention” and “sustained attention” (Sparby & Sacchet, 2024, p. 1383). More literal translations are “conception” (*vitakka/vitarka*) and “examination” (*vicāra*). This means that to focus on a chosen object, in the first place, a general notion of this object is needed to direct attention to it. Then, it is necessary to examine or investigate it more closely, with interest and curiosity, in order to sustain attention on it. These two factors are conceptual in nature, but paradoxically they may serve to enhance the direct sense-perception of an object like the breath. This ancient classification may serve to reassert the paramount importance of the clarity of intentions and guiding instructions for the effective practice of mindfulness meditation. The next factors, “joy” conveying a sense of energy, and “happiness” a sense of tranquility, show that, at a certain stage of progress, the experience of concentration on one task is a deeply enjoyable one. The fifth factor is that of mental unification or “one-pointedness.”

Again, like in the contemporary psychology of flow, such endogenous enjoyment is conceived to derive from the mastery of a skilled activity (Csikszentmihalyi, 2008, pp. 49–53). It is therefore to be radically differentiated for an exogenous pleasure that is the object of the first obstacle to concentration, sensual desire, or craving for instant gratification. Backed up with such a psychoeducation, mindfulness basic training may therefore empower students to overcome the addiction to quick rewards brought by digital technologies, acquire self-regulation skills, and find joy in the disciplined yet enthusiastic effort of learning.

Csikszentmihalyi’s psychology of flow experience may be seen as the re-actualization of Aristotle’s views on *happiness as an activity*, and not a passive state. He even conceded (2008, p. 1) that “We do not understand what happiness is any better than Aristotle did.” According to Aristotle (1996, *Nicomachean Ethics* X, 1177a18–1178b7), the most satisfying activity, which, in its own process, contains its own reward, was conceived as that of the intellect, or contemplation (in its ultimate dimension, an intellectual intuition). And life lived in accordance with it was considered the happiest. Such ancient wisdom tradition finds contemporary echoes in the mindful movement’s notions of the “being mode” (Segal et al., 2013, pp. 72–74), of “resting in awareness itself” (Kabat-Zinn, 2013, p. 30), content to just be. The important difference with the ancient West though, in that such experiences of pure presence, so to speak, are explicitly developed by relying upon the refinement of bodily awareness (and not by discarding the body). These experiences, to be discovered by oneself, form in the end the horizon of a mindfulness-based higher education, here envisaged according to the joy of learning, learning to live according to what, in us, is the best: mindful living.

## Conclusion: Mindful Education as the Formation of Attention

In this article, we have intended to gather different disciplinary perspectives on mindfulness for higher education, from the languishing to the flourishing of students, considering issues in mental health and special care for vulnerabilities; listening to their voices and deep concerns; envisioning then the mindful way back from information to knowledge, to wisdom, and to Life; conceptualizing mindfulness (especially with MBCT’s 3SBS) as the guiding thread for this path; and as the means for finding joyful flow in learning. This theoretical essay is meant to restitute the larger picture that is often missing (and thus leading to criticism of MBPs, whether these are fair or unfair), and to highlight the main articulations between mindfulness and higher education. Our conceptual synthesis invites verification through future evidence-based research (in particular, its proposal to introduce largely the 3SBS into higher education), but it may already serve as a compass guiding the implementation of mindfulness training for higher education, by understanding it mainly as the holistic formation of attention. Further advancement will also require the full development of an *ethics of attention*, tackling the fundamental questions of the redirection of desire, value, and interest in our contemporary societies.

## Declarations

### Use of Artificial Intelligence

We did not use AI when writing this manuscript.
